# Examining the function of macrophage oxidative stress response and immune system in glioblastoma multiforme through analysis of single-cell transcriptomics

**DOI:** 10.3389/fimmu.2023.1288137

**Published:** 2024-01-11

**Authors:** Jin Xing, Huabao Cai, Zhiheng Lin, Liang Zhao, Hao Xu, Yanbing Song, Zhihan Wang, Chaobo Liu, Guangdong Hu, Jiajie Zheng, Li Ren, Zilong Wei

**Affiliations:** ^1^ Department of Neurosurgery, Shanghai Pudong Hospital, Fudan University Pudong Medical Center, Shanghai, China; ^2^ Department of Neurosurgery, First Affiliated Hospital of Anhui Medical University, Hefei, China; ^3^ Shandong University of Traditional Chinese Medicine, Jinan, China

**Keywords:** glioblastoma, macrophages, immune mechanism, oxidative stress response, single-cell transcriptome sequencing

## Abstract

**Background:**

Glioblastoma (GBM), a prevalent malignant neoplasm within the neuro-oncological domain, has been a subject of considerable scrutiny. Macrophages, serving as the principal immunological constituents, profoundly infiltrate the microenvironment of GBM. However, investigations elucidating the intricate immunological mechanisms governing macrophage involvement in GBM at the single-cell level remain notably limited.

**Methods:**

We conducted a comprehensive investigation employing single-cell analysis, aiming to redefine the intricate cellular landscape within both the core and peripheral regions of GBM tumors. Our analytical focus extended to the profound study of macrophages, elucidating their roles within the context of oxidative stress, intercellular information exchange, and cellular trajectories concerning GBM and its assorted subpopulations. We pursued the identification of GBM prognostic genes intricately associated with macrophages. Utilizing experimental research to investigate the relevance of MANBA in the context of GBM.

**Results:**

Our investigations have illuminated the central role of macrophages in the intricate interplay among various subpopulations within the GBM microenvironment. Notably, we observed a pronounced intensity of oxidative stress responses within macrophages when compared to their GBM counterparts in other subpopulations. Moreover, macrophages orchestrated intricate cellular communication networks, facilitated by the SPP1-CD44 axis, both internally and with neighboring subpopulations. These findings collectively suggest the potential for macrophage polarization from an M1 to an M2 phenotype, contributing to immune suppression within the tumor microenvironment. Furthermore, our exploration unearthed GBM prognostic genes closely associated with macrophages, most notably MANBA and TCF12. Remarkably, MANBA appears to participate in the modulation of neuroimmune functionality by exerting inhibitory effects on M1-polarized macrophages, thereby fostering tumor progression. To bolster these assertions, experimental validations unequivocally affirmed the promotional impact of MANBA on GBM, elucidated through its capacity to curb cell proliferation, invasiveness, and metastatic potential.

**Conclusion:**

These revelations represent a pivotal step towards unraveling the intricate immunological mechanisms governing the interactions between macrophages and diverse subpopulations within the GBM milieu. Furthermore, they lay the foundation for the development of an innovative GBM prognostic model, with MANBA at its epicenter, and underscore the potential for novel immunotherapeutic targets in the ongoing pursuit of enhanced treatment modalities for this formidable malignancy.

## 1 Introduction

Glioblastoma (GBM) is the most prevalent and aggressive primary intracranial cancer in humans (1), comprising approximately 57% of all gliomas and 48% of all primary neurological malignancies. It represents one of the most advanced and pernicious forms of brain tumors (2) GBM can be classified into IDH wild-type and IDH-mutant subtypes. Histologically, both subtypes exhibit features characteristic of high-grade astrocytomas. The IDH wild-type GBM is predominantly primary, while the IDH-mutant GBM is often associated with a history of low-grade astrocytoma (3–5). Currently, standard treatments for GBM primarily involve surgical intervention, followed by adjuvant radiotherapy and chemotherapy. Additionally, there are emerging modalities such as targeted immunotherapy and electric field therapy. Unfortunately, due to its malignant proliferation, infiltration into brain parenchyma, and resistance to treatment, patients often experience unfavorable outcomes, with a one-year survival rate of 40% and a five-year survival rate of 5.6% (6, 7). In recent years, immunotherapy has achieved significant success in improving the prognosis of many cancer patients, emerging as a new ray of hope for numerous individuals battling cancer. The immunotherapeutic strides achieved in cancer have rendered immunotherapy particularly appealing for GBM. Presently, immunotherapeutic approaches for GBM encompass CAR-T cells, oncolytic viruses, cancer vaccines, and immune checkpoint inhibitors. Unfortunately, the relative immaturity of GBM immunotherapy persists due to the “immune privilege” of the brain and the immunosuppressive microenvironment within GBM (8, 9). Consequently, investigating the immune-related mechanisms of GBM and developing novel immunotherapeutic approaches to enhance the prognosis of GBM patients assumes paramount importance.

Macrophages, as human immune regulatory effector cells, play a crucial role in tumor occurrence and progression. In the tumor microenvironment, macrophages make up more than 50% of infiltrating immune cells (10), which can promote vascular growth, tumor proliferation, metastasis, and drug resistance (11). However, macrophages are also the main immune cells that infiltrate the GBM microenvironment. Under normal circumstances, peripheral macrophages find it challenging to penetrate the central nervous system (CNS) due to the presence of the blood-brain barrier (BBB). However, GBM can damage and induce the BBB, which allows macrophages in the peripheral blood to cross the BBB and accumulate in the tumor microenvironment (12). Relevant research has demonstrated the crucial role of communication between tumor cells and macrophages in the malignant progression of GBM. Their functions include supporting angiogenesis, nurturing tumor stem cells, and promoting an immunosuppressive tumor microenvironment (13, 14). However, other studies have found that, due to diverse inducing factors, macrophages within tumors can undergo differentiation and mutual transformation between M1/M2 phenotypes. M1 macrophages are associated with tumor suppression, whereas M2 macrophages may facilitate tumor growth (12, 15). This suggests that the specific impact of macrophages on GBM could be bidirectional, depending on their differentiation direction. Moreover, these effects may be realized through specific pathways and mechanisms within GBM. Modulating or enhancing certain pathways or mechanisms could potentially have therapeutic implications for GBM.

Redox homeostasis is fundamental to maintain normal cell function and ensure cell survival (16). However, a high oxidative stress state is often present in tumor cells (17). Excessive production of ROS caused by oxidative stress imbalance may induce somatic cell mutations (18) and destroy nuclear DNA and mitochondrial DNA, thereby increasing the risk of cancer (19, 20). However, some researches show that, ROS may participate in inducing the polarization of macrophages from the phenotype M1 to M2 except for its involvement in tumorigenesis, thus causing suppressive tumor immune microenvironment (21). However, clinical immunotherapy trials for GBM, including vaccines, adoptive cellular therapy and immune checkpoint blockade, have fewer actual benefits to patients (22, 23). Therefore, we suspect that the oxidative stress response of macrophages may be involved in the immunosuppression in the GBM microenvironment.

In this investigation, we showcase the glioblastoma multiforme (GBM) core and its surrounding tissues’ associated cells. We delve into the response of macrophages to oxidative stress and associated transcription factors. Furthermore, we outline the trajectory relationship between macrophages and other cells and establish a GBM prognostic model that is associated with macrophage genes. These findings will enable us to gain deeper insight into the immune interaction mechanism between macrophages and GBM, thereby facilitating the development of more targeted immunotherapy for GBM and the creation of a prognostic model.

## 2 Methods

### 2.1 Data download and processing

Single-cell SRA file data of the tumor core and peritumoral tissue of four GBM patients were downloaded fromGene Expression Omnibus (GEO) database (https://www.ncbi.nlm.nih.gov/geo/), containing eight samples (SRR13194337-SRR13194344). The SRR files were converted into fastq format and the downstream input files were generated with cellranger (7.0.1) to compare GRCh38 human reference genomes under default settings. Gene expression quantification RNA-Seq (HTSeqFPKM) and clinical data of GBM were downloaded from TheCancer Genome Atlas Program(TCGA) website https://www.cancer.gov/ccg/research/genome-sequencing/tcga). The data were extracted and standardized using the R software (R 4.1.1).

### 2.2 Quality control

To ensure the accuracy of downstream analysis, we utilized the R package DoubletFinder to exclude capsule cells and filter out low-quality cells. The filtering criteria used were: 1) The total number of genes transcribed per cell is below 80,000. 2) The total number of genes detected per cell is below 8,000. 3) The proportion of mitochondrial gene count is less than 20%. 4) The proportion of red blood cell gene count is less than 5%.

### 2.3 Dimensionality reduction, clustering and annotation of data

The cells that had been filtered were normalized through the application of the “NormalizeData” function of the Seurat R package. To identify the top 2000 highly variable genes, the “FindVariableFeatures” function was utilized based on dispersion degree and mean expression. The caladata function was used to conduct standardized scaling. The CellCycleFeatures function was utilized to calculate cell-cycle effects. Batch effect was eliminated using the Harmony R package. Principal component analysis/dimensionality reduction was performed using the RunPCA function based on the expression of the top 2,000 hypervariable genes, and clustering was done using the FindNeighbors and FindClusters functions. The clustering of groups was annotated with the help of the “singleR” R package and related literature data. The differentially expressed genes with other different clusters were calculated using the FindAllMarkers function.

### 2.4 Oxidative stress pathway scores

The R package AUCell was employed to score the activities of various cellular oxidative stress pathways. The scores were compared between the most active macrophages and other cells, and validated using the PercentageFeatureSet and AddModuleScore functions.

### 2.5 Macrophage ratio and related transcription factors (SCENIC analysis)

To investigate the distribution of cells within the tumor core and peritumoral tissue, we utilized R software for visualization purposes. Furthermore, we aimed to identify the macrophage-associated transcription factor (TF) regulatory network, which exhibited the most significant changes in proportion. This was accomplished using the pyscenic method. Initially, GRNBoost was utilized to identify potential targets for each TF. Subsequently, potential direct binding targets were chosen based on DNA-motif analysis and cellular regulons activities were scored using AUcell. Finally, the top five with the highest scores were selected for discussion of their expression in different cells.

### 2.6 Differential genes and enrichment analysis

We identified differentially expressed genes (DEGs) in various kinds of cells. DEGs must be detected in 25% of the cells and P<0.01, false discovery rate (FDR)<0.05, | logFCfilter |> 1. Kyoto Encyclopedia of Genes and Genomes (KEGG) enrichment analysis was performed.

### 2.7 Cell-cell interaction analysis

All intercellular interactions were analyzed using the R package cellchat, which allowed us to predict the potential interaction strength of macrophages with other cells based on the mean expression number of receptors and ligands. Different incoming and outgoing signals of various cells were visualized to gain a better understanding of the complex intercellular communication networks in the GBM tumor microenvironment.

### 2.8 Cell trajectory analysis

We conducted cell trajectory analysis for different types of cells using the velocyto method to study their transformation and evolution process. Through dimensionality reduction and cluster analysis of the data, we determined the cell-cell distance and RNA transformation rate to indicate the direction of cell differentiation. Furthermore, we employed the paga method to investigate the confidence size of different intercellular trajectories. Finally, we verified cellular differentiation lineage construction and pseudo-time inference using cell clustering and spatial dimensionality reduction information through the slingshot tool.

### 2.9 High-throughput weighted co-expression network analysis

HdWGCNA is a systematic biological analysis method that can be used to describe gene-associated patterns and identify co-expressed gene modules. By hdWGCNA analysis of eight samples, we obtained gene modules related to macrophages. They were also scored by KME and visualized in terms of their expression in various kinds of cells. Gene modules associated with macrophages were further screened. The top 25 genes were selected for network analysis according to their scores of KME. Of which the top 25 genes were picked up for the network analysis.

### 2.10 The clinical relevance and independent prognostic assessment

GBM were excluded. Then, the intersected genes were merged with standardized GBM clinical data. The univariate Cox proportional hazards regression analysis was performed using the ‘coxph’ function from the R package ‘survival,’ followed by validation using least absolute shrinkage and selection operator (LASSO)-penalized Cox regression to prevent overfitting issues. Subsequently, a multivariate Cox proportional hazards regression analysis was conducted to identify two macrophage-related differential genes (MR-DEGs) associated with prognosis. To compute the risk score of each sample (Risk Score = Xλ, where Xλ represents the relative expression levels of prognostically relevant genes and coefλ represents the coefficients), we stratified the samples into high and low-risk groups based on the median score. Subsequently, we employed Principal Component Analysis (PCA) to examine the distribution patterns within these groups. We conducted an investigation into the survival outcomes and expression profiles of MR-DEGs between the high and low-risk groups. The results of this analysis were visually represented using Kaplan-Meier survival curves. Furthermore, the specificity and sensitivity of our risk score-based prediction were assessed through time-dependent receiver operating characteristic (ROC) curves. In order to investigate the correlation between clinical factors and the risk score, as well as to assess whether it can serve as an independent prognostic indicator, we incorporated demographic data including age, gender, and ethnicity from our sample cohort. We then compared the differences in these factors between the high and low-risk groups. To construct a predictive nomogram for the prognosis of GBM patients and validate its performance, we employed the R package ‘rms’. This nomogram will enable us to estimate patient outcomes based on a combination of the risk score and clinical variables, providing a comprehensive tool for prognostic assessment in GBM.

### 2.11 The analysis of MR-DEGs in relation to macrophages and associated risk

Through the application of the xCell and CIBERSORT deconvolution algorithms, we have unveiled the landscape of immune infiltration among patients in both high and low-risk groups. Furthermore, we have conducted a meticulous calculation to determine the associations between immune cell populations and their respective risk scores, as well as the MR-DEGs. Subsequently, based on our findings, we embarked on an exploration and visualization of the interrelationships involving M1 macrophages, MANBA expression, and risk scores. This multifaceted analysis not only deepens our comprehension but also provides a visual representation of these intricate connections. In addition, we harnessed the “ESTIMATE” algorithm to calculate the stromal score, immune score, and the overall microenvironment score for both high and low-risk groups. Our investigation also extended to the examination of their correlations with risk scores and MNABA expression, thus shedding light on the complex interplay between the tumor microenvironment, risk assessment, and MNABA expression.

### 2.12 Differential gene enrichment analysis in high and low-risk groups

We obtained differential gene expression profiles between high and low-risk groups using the R package “limma,” with the following filtering criteria: |log2 Fold Change| > 1 and a false discovery rate (FDR) adjusted threshold of P.adj < 0.05. Subsequently, we performed KEGG enrichment analysis and Gene Ontology (GO) enrichment analysis, including biological processes, cellular components, and molecular functions, using the “clusterProfiler” R package. Furthermore, we employed the Gene Set Enrichment Analysis (GSEA) algorithm to analyze the expression gene sets from KEGG databases (c2.cp.kegg.v7.5.1.symbols.gmt) that were collected as marker gene sets for low-risk and high-risk populations. Statistically significant results were defined as FDR < 0.05.

### 2.13 Cell lines and cultures

Human glioma cell lines U87-MG and LN227 used in this study were purchased from the Cell Center of Shanghai Institutes for Biological Sciences (Shanghai, China). Glioma cells were cultured in DMEM supplemented with 10% fetal bovine serum (FBS; WISENT, Canada) and antibiotics (1% penicillin/streptomycin, Gibco, USA) in a humidified atmosphere of 95% air and 5% CO_2_ at 37 °C. Cells in this study were passaged when they reached ~70% confluency.

### 2.14 Transfection

Small interfering RNA (siRNA) used in this study was synthesized by GenePharma. The procedure of transfection was performed according to the instructions of lipofectamine 3000(Invitrogen, USA).

### 2.15 Colony formation assay

Treated glioma cells were seeded into 6-well plates at a density of 1000 cells/well. After 12 days of culture, cells were fixed by 4% paraformaldehyde and stained with crystal violet for 30 minutes, then photographed and counted. Each experiment was repeated for three times.

### 2.16 Wound healing assay

Wound healing assay were operated 48 hours after transfection. U87-MG and LN227 cells were seeded into each well until confluence, 200μl pipette tip were used to draw a straight line. Then the supernatant in each well was replaced. After the cells were cultured in serum-free PRMI-1640 for 0, 48h, migration imagines were captured. Each experiment was repeated for three times. The cell-free areas were calculated and quantified by ImageJ software.

### 2.17 Transwell assay

Transwell chambers with a membrane pore size of 8μm (Corning, USA) were coated with (transwell invasion assay) or without (transwell migration assay) Matrigel (BD Biosciences, USA). A total of 3×10^4^ cells were seed into the upper chambers with serum-free medium, whereas medium containing 10% FBS was used in the lower chamber. After incubation for 48h, the cells on the bottom of chamber were fixed, stained, and counted by inverted microscope.

## 3 Results

### 3.1 GBM cell classification (or GBM single cell data analysis)

**Figure 1:**
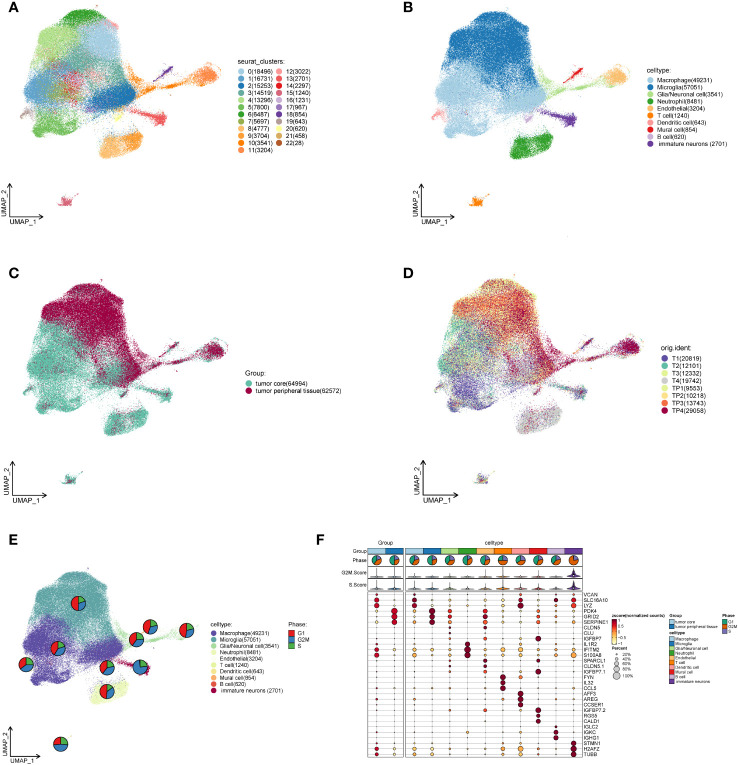
Single cell analysis of cell subsets in the GBM core and surrounding tissues. **(A)**. The cells of the tumor core and peritumoral tissues are gathered to 23 clusters in four GBM patients. **(B)**. Macrophage, Microglia, Glia/neuronal cell, Neutrophil, Endothelial, T cell, Dendritic cell, Mural cell, B cell and immature neurons are annotated based on different cell surface genes. **(C)**. Overall distribution of the peritumoral tissue and tumor core tissue samples. **(D)**. Distribution of tumor core and peritumoral tissues in four GBM patients. **(E)**. The proportional size of different stages of G1 and G2MS in each cell subset of GBM. **(F)**. MARKER gene expression in GBM core and peritumoral tissues as well as each cell subset.

We downloaded the single cell data (T1: 21764, T2: 14612, T3: 14070, T4: 24336) in the tumor core and single cell data (TP 1: 10746, TP 2: 11454, TP 3: 15998, TP3: 41926) in the peritumoral tissue of four GBM patients from the Geo database. The capsule cells were removed through R package DoubletFinder to obtain the single cell data (T1: 21462, T2: 13072, T3: 12644, T4: 24336) in the tumor core and the single cell data (TP 1: 10746, TP 2: 10543, TP 3: 14190, TP 4: 29074) in the peritumoral tissue (**Supplementary Figure 1**). It was filtered using the R software (**Supplementary Figure 2A**) to eventually obtain 127,566 single cell data. By cell staging test, we found that the points in PCA map were more concentrated (**Supplementary Figure 2B**). It indicates that cell staging is smaller than our results. We selected the top 2,000 diversity genes (**Supplementary Figure 2C**) and performed the dimensionality reduction per diversity genes by RunPCA (**Supplementary Figure 2D**). The first 30 dimensions were finally picked up to visualize (**Supplementary Figure 2D**). By clustering, we divided all cells into 23 clusters (**Figure 1A**) which were further annotated as 10 categories of cells by R package singleR and literature review (**Figure 1B**). The type and number of cells included macrophage (49,231), microglia (57,051), glia/Neuronal cell (3,541), neutrophil (8,481), endothelial (3,204), T cell (1,240), dendritic cell (643), mural cell (854), B cell (620) and immature neurons (2701), respectively. In order to explore the differences in the cell composition in the tumor core and peritumoral tissue, we visualized the cells of different samples sources (**Figures 1C, D**), and found that there was significant difference in cell distribution in the tumor core and peritumoral tissue. The cells around the tumor tissue were mostly microglia, glia/neuronal cell, endothelial and mural cell while the cells in the core were mainly macrophage, neutrophil, T cell, dendritic cell, B cell and immature neurons. We discovered that immune cells aggregated more in the tumor core tissue and the macrophages were particularly significant. Then we studied the mitotic cycle of various types of cells (**Figure 1E**), and found that immature neurons belonged to the tumor core cells and the G2M stage accounted for more, indicating that its metabolism, division and so on life activities were vigorous. We further explored marker genes with higher average expression number and percentage of expressed cells among different cells (**Figure 1F**), and found that in the tumor core tissue, the genes VCAN, SLC16A10 and LYZ were significantly coexpressed in macrophages, dendritic cells and immature neurons; genes IFITM2 and S100A8 were significantly expressed in neutrophils; and genes H2AFZ and TUBB were significantly expressed in immature nerve cells. In the peritumoral tissue, genes PDK 4 and SERPINE1 were significantly expressed in microglia, glia/neuronal cell, endothelial and mural cell, particularly evident in the microglia.

### 3.2 Oxidative stress pathway activity analysis

**Figure 2:**
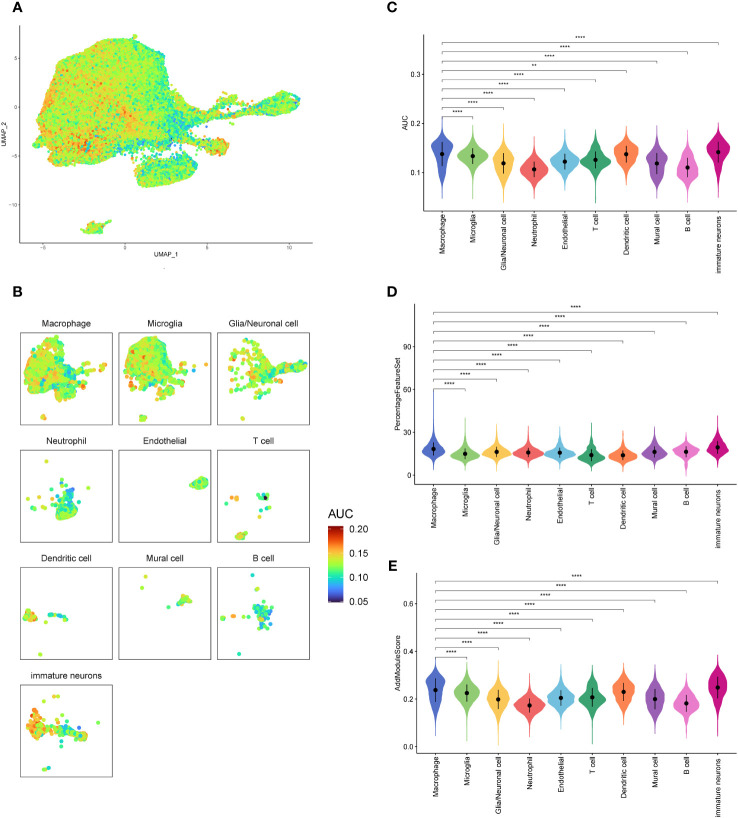
Oxidative stress pathway scores of GBM cell subsets. **(A, B)**. AUCELL algorithm score distribution diagram of oxidative stress pathway activity. **(C)**. Difference in AUCELL scores of oxidative stress between the macrophages and other cell subsets. **(D)**. Difference in oxidative stress scores by PercentageFeatureSet function between the macrophages and other cell subsets. **(E)**. Differences in oxidative stress scores by AddModuleScore function between the macrophages and other cell subsets. **p<0.01; ****p<0.0001.

In order to investigate the activity of oxidative stress pathways in different cells of GBM, we scored them using the R package AUCell (**Figures 2A, B**). And we found that the oxidative stress activity of macrophages was significantly increased. Therefore, we are interested in the specific role of oxidative stress in GBM. Then we compared the oxidative stress activity scores (**Figure 2C**) between the macrophages and other cells, which were validated by PercentageFeatureSet and AddModuleScore functions (**Figures 2D, E**), and found that the oxidative stress scores of macrophages calculated by the three methods were higher and more significantly different than most other cells. This may suggest that macrophages involve in oxidative stress response and play an important role in the occurrence and development of the tumor.

### 3.3 Subset proportion and identification of related transcription factors

**Figure 3:**
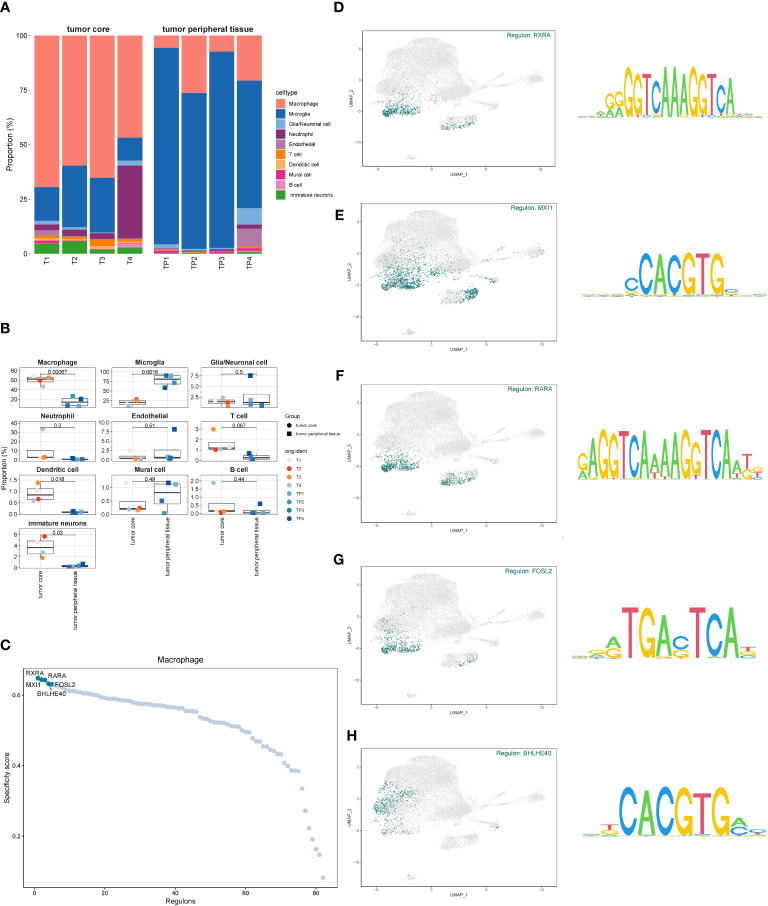
Proportion of each cell subset of GBM and transcription factor identification. **(A, B)**. The proportion and difference of each cell subset between the GBM core and surrounding tissues. **(C)**. Macrophage-associated oxidative stress pathway transcription factor scores. **(D–H)**. Expression of the top five scored transcription factors in each cell subset.

By comparing the proportion of cell types in the GBM core samples to the peripheral samples (**Figures 3A, B**), we discovered that the proportion of macrophages in the tumor core tissue was significantly elevated. In contrast, the proportion of microglia was significantly reduced, suggesting that these two types of cells may have opposite effects on tumor progression. In addition, the proportion of dendritic cells and immature neuronal cells was also relatively increased in the tumor core tissue, which may indicate a positive implication for tumor development. However, glia/neuronal cell, endothelial, neutrophil, T cell, mural cell and B cell showed a similar proportion in the tumor core and the peritumoral tissue. The difference was not statistically significant (P>0.05). We further studied the transcription factors of the macrophage-related oxidative stress pathways (**Figure 3C**) and selected the top five with highest scores, namely RXRA, RARA, MXI 1, FOSL 2 and BHLHE40. In order to investigate the relationship between these five transcription factors and GBM-related cells, we further visualized the cell subsets expressed by these five transcription factors (**Figures 3D-H**), and found that they were transcriptionally expressed in the tumor core tissue, mainly in the macrophage group and also partially distributed in the neutrophils.

### 3.4 Enrichment analysis of differential genes

**Figure 4:**
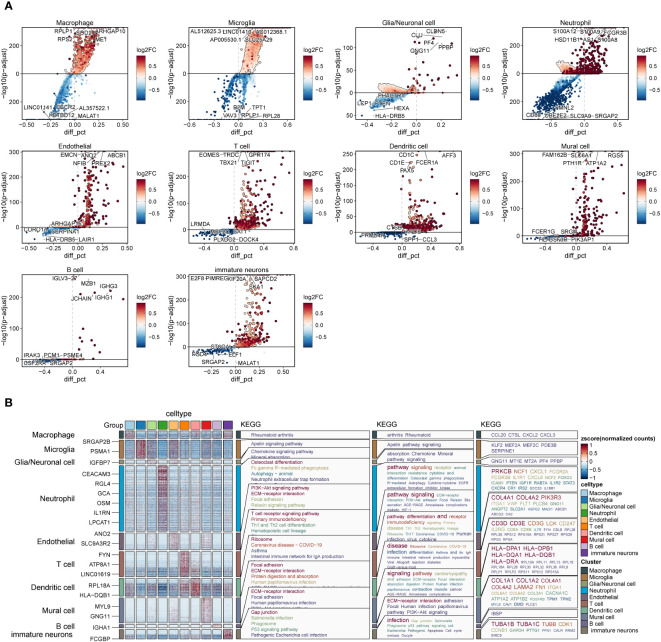
Differential genes and enrichment analysis in each cell subset of GBM. **(A)**. Differentially expressed genes in each cell subset of GBM. **(B)**. KEGG enrichment analysis of differential genes in each subset of GBM.

We screened the differential genes for different types of cells (**Figure 4A**) and showed the top five genes with the highest and lowest scores, respectively. Furthermore, KEGG enrichment analysis was performed for each type of cells using these differentially expressed genes (**Figure 4B**). We found that arthritis rheumatoid acted significantly in macrophages, while apelin signaling pathway enrichment was significant in microglia. We speculate that this may be an antagonistic effect of microglia on the oxidative stress response of macrophages, which has an inhibitory effect on the tumor progression.

### 3.5 GBM intercellular interaction analysis

**Figure 5:**
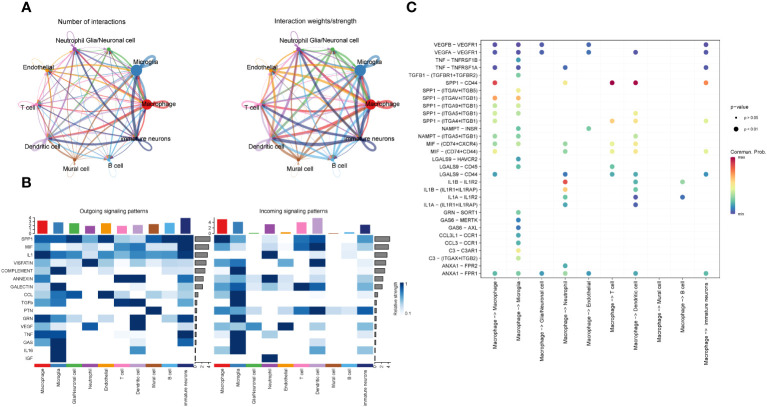
Information interaction among each cell subset of GBM. **(A)**. Diagram of the cell interaction trajectory of each cell subset of GBM, with the number of cell interaction species indicated on the left and the cell interaction intensity on the right. **(B)**. Cell transmission signal factors of each subset, efferent on the left and afferent on the right. **(C)**. Bubble chart of the interaction between the macrophages and receptor-ligand pairs in each cell subset.

In order to study the interaction among different cells in GBM, we analyzed it using the R package cellchat (**Figure 5A**) and discovered that the interaction types and interaction intensity of macrophages and other cells were rather high. Furthermore, we summarized the afferent and efferent signal factors of different cell subsets in GBM (**Figure 5B**) and found that the overall intensity of macrophages receiving and sending signal factors was higher than that of other cells, followed by microglia and immature nerve cells. Based on these signaling patterns, we focused on the interaction between interligand-receptor pairs of macrophages and other cell subsets (**Figure 5C**) and discovered that the SPP1-CD44 receptor-ligand pair had a higher intercellular communication within macrophages. It was also present in the interaction between the macrophages with T cells, dendritic cells and immature immune cells. In addition, we discovered that macrophages and microglia had the highest number and intensity of receptor-ligand interactions. We believe that there may be some antagonistic links between the two cells. In addition, we found that VEGFB-VEGFR1 and ANXA1 FPR1 receptor-ligand pairs appeared more frequently when macrophages interacted with a variety of cells.

### 3.6 GBM-associated cell trajectories

**Figure 6:**
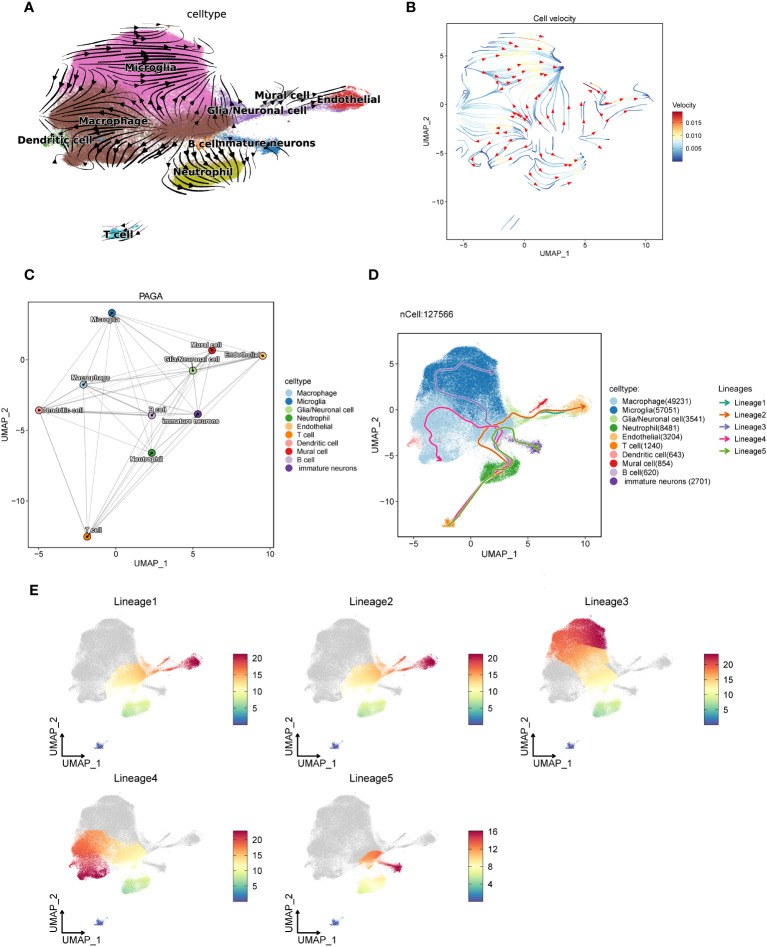
The trajectory of each subset of GBM cells is analyzed using three different algorithms. **(A, B)**. The trajectory direction map among GBM cells calculated by the velocyto method, with the tip representing the direction and the trajectory color representing the velocity scale. **(C)**. The confidence of the trajectory of each subset calculated by the paga method. The thicker the line is, the closer the correlation will be. **(D)**. The 5 trajectory charts are calculated in each cell subset by slingshot methods. **(E)**. Cell subsets pass through the five cell trajectories, with red indicating close to the end point and blue close to the start point.

In order to understand the trajectory relationship between the macrophages and other GBM cell subsets, we conducted the trajectory analysis for GBM core tissue and peripheral tissue cells by velocyto method (**Figures 6A, B**), and found that macrophages seemed to be in the center of the trajectory and progressed to the surrounding subsets. It seems to confirm the results of the macrophages interacting with a variety of cells described previously. We further explored the confidence size of the cell trajectory among the subsets by paga method (**Figure 6C**), and found that the macrophages were closely related to the other subsets. We also verified the cell-to-cell trajectory by slingshot (**Figures 6D, E**), and found that the five cell trajectories reached the macrophages and then flowed to other cell subsets. The macrophages can be the starting point of the bifurcation of the cell trajectory. We speculate that it may be related to the interactions between the macrophages and other cells.

### 3.7 Weighted co-expression network analysis

**Figure 7:**
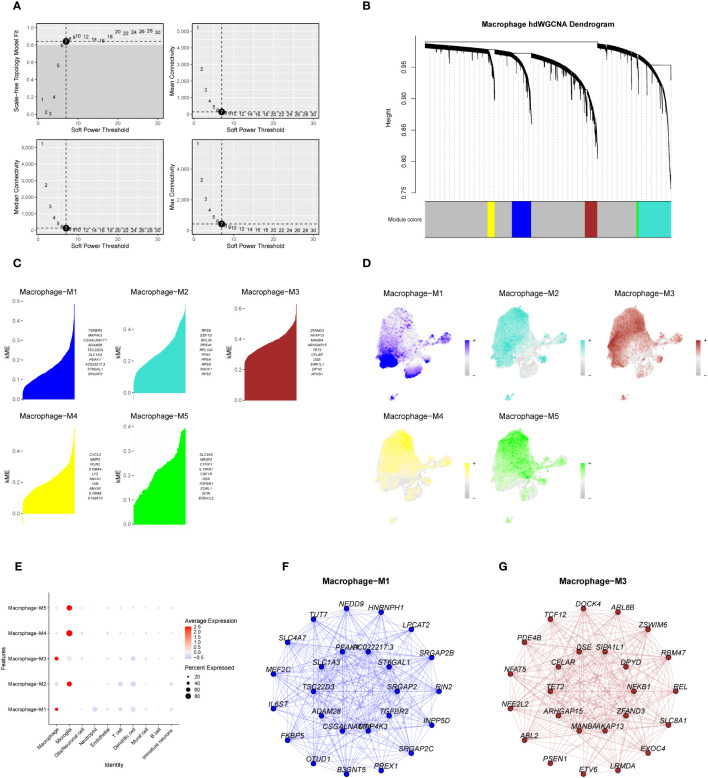
The weighted co-expression network analysis of macrophage-related genes and the establishment of gene modules. **(A)**. Macrophage-associated WGCNA analysis of data from eight samples, with a soft threshold set to 7. **(B)**. Dendrogram of the five non-gray macrophage gene modules. **(C)** KME scores of the five gene modules and the top ten genes with a higher score. **(D, E)**. Expression of genes within the five gene modules in each cell subset of GBM. **(F, G)**. Network map of the top 25 genes within the M1 and M3 gene modules.

Macrophage-related modules were obtained through WGCNA analysis of data from eight samples. A soft threshold was set to 7 (**Figure 7A**), minimum number of module genes to 100, depth in split to 2, and combined similarity less than 0.5. We obtained a total of 5 non-gray gene modules M1-M5 (**Figure 7B**);, completed the KME scores for the five gene modules to determine the highly connected genes within the modules (**Figure 7C**), and found that the M3 module had the highest score. Through further visualizing the expression of five gene modules in cell subsets (**Figure 7D**), it was found that the M1 gene module was mostly expressed in the tumor core tissue and concentrated in the macrophage subsets. We speculate that the M1 and M3 gene modules are related to macrophages. We verified the relationship between the five gene modules and each cell subset of GBM. The results are consistent with our hypothesis (**Figure 7E**). We screened the top 25 genes (**Figures 7F, G**) of the M1 and M3 gene modules based on the KME scores for the next survival prognostic analysis.

### 3.8 Prognostic analysis

**Figure 8:**
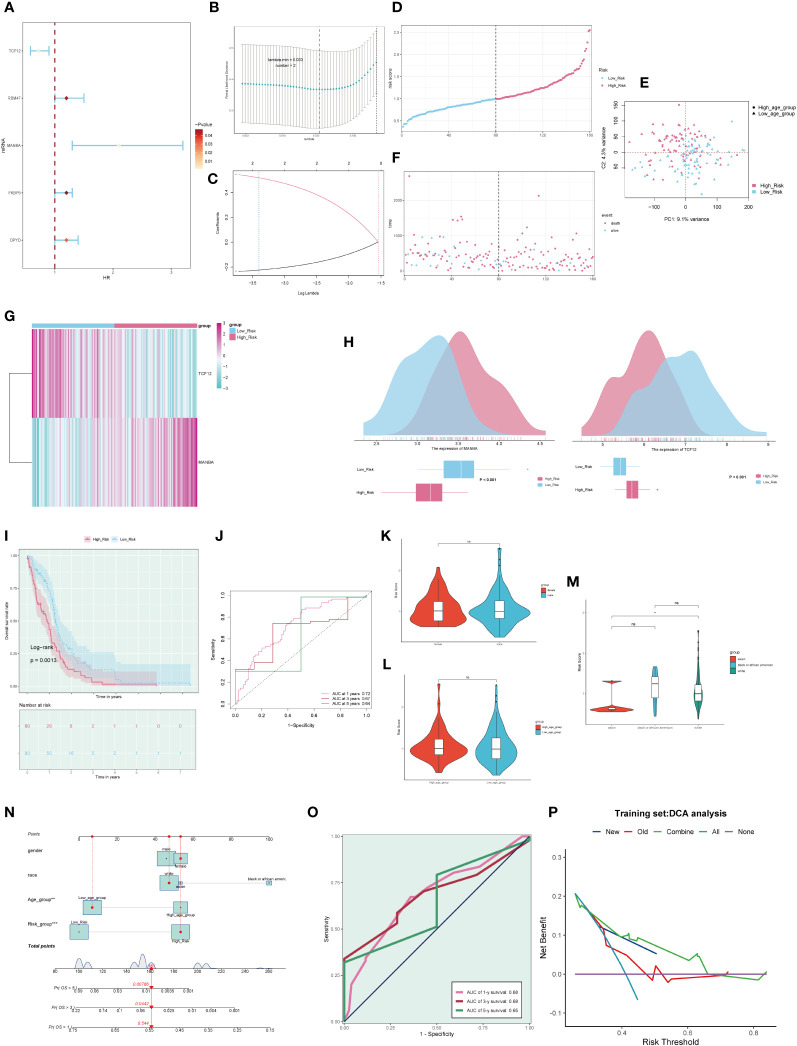
Clinical relevance of genes and independent prognostic analysis. **(A)**. Forest plot of five macrophage-related genes, highlighting genes associated with the prognosis of GBM through univariate Cox analysis. **(B)**. A coefficient spectrum distribution of the five genes using LASSO analysis. **(C)**. Parameter selection in the optimal cross-validated LASSO regression. **(D)**. Patients were stratified into high and low-risk groups based on their risk scores. **(E)**. Distribution of patients in the high and low-risk groups. **(F)**. Expression profiles of prognostic-related genes in the high and low-risk groups. **(G)**. Heatmap depicting the distribution of prognostic-related genes. **(H)**. Differential expression of prognostic-related genes in the high and low-risk groups. **(I)**. Kaplan-Meier survival analysis curves for the high and low-risk groups. **(J)**. Time-dependent ROC curves with AUC values of 0.723, 0.707, and 0.618 for 1-year, 3-year, and 5-year intervals. **(K-M)**. Analysis of the correlation between risk scores and factors such as gender, age, and ethnicity. **(N)**. Survival curve plots for GBM patients at 1-year, 3-year, and 5-year intervals. **(O)**. Time-dependent ROC curve plots with AUC values of 0.68, 0.68, and 0.65 for 1-year, 3-year, and 5-year intervals. **(P)**. Decision curve analysis plot used to assess independent prognostic factors. ns, no statistical difference; *p<0.05.

By intersecting the top 25 genes from the M1 and M3 modules with differentially expressed genes (DEGs) obtained from the TCGA database for Glioblastoma Multiforme (GBM), we collectively acquired 50 Most Relevant Differentially Expressed Genes (MR-DEGs). Subsequently, employing univariate Cox regression analysis, we meticulously sieved through these genes and identified 5 MR-DEGs that displayed prognostic associations(**Figure 8A**). To further ascertain their robustness, we conducted LASSO Cox regression analysis, the results of which affirmed the stability and reliability of these genes (**Figures 8B, C**). Ultimately, through a multivariate Cox regression analysis, we conclusively identified two MR-DEGs significantly associated with prognosis: the high-risk gene MANBA and the low-risk gene TCF12. We computed risk scores for each sample, stratifying them into high and low-risk groups based on the median score (**Figure 8D**). Furthermore, we utilized Principal Component Analysis (PCA) to validate the distinct distribution of patients from different risk groups along two divergent axes (**Figure 8E**). We evaluated the relationship between survival status and survival time among high and low-risk groups (**Figure 8F**). Notably, we observed that with increasing risk scores, there was a greater concentration of deceased patients, indicating a correlation between higher risk scores and poorer survival outcomes. Subsequently, we delved deeper into the expression patterns of the two prognostically relevant MR-DEGs within the high and low-risk groups (**Figures 8G, H**). It was discerned that MANBA exhibited elevated expression levels in the high-risk group, while TCF12 demonstrated higher expression levels in the low-risk group, with statistically significant differences noted. The Kaplan-Meier survival curve (**Figure 8I**) clearly illustrates that the survival rate in the high-risk group is consistently lower than that in the low-risk group at different time points, with a significant statistical difference of P < 0.01. This underscores the meaningfulness and clinical relevance of our results. The results from the ROC curves for the 1-year, 3-year, and 5-year survival predictions (**Figure 8J**) yield respective areas under the curve (AUC) values of 0.72, 0.67, and 0.64. These findings signify that our predictive model exhibits stability and excellence.

Furthermore, we conducted an in-depth examination of the correlations between various clinical factors and risk scores (**Figures 8K-M**). Subsequently, based on the risk scores and clinical factors, we constructed a nomogram plot to enhance the prediction of survival rate changes over 1 year, 3 years, and 5 years for different patients (**Figure 8N**). To further assess the predictive performance of our model, we generated ROC curves for each year’s survival prediction (**Figure 8O**). The results revealed AUC values of 0.68, 0.68, and 0.65 for 1 year, 3 years, and 5 years, respectively, indicating favorable sensitivity and specificity of our predictive model. Additionally, decision curve analysis (**Figure 8P**) was employed to compare the clinical net benefits of different prediction models. The results clearly demonstrate that the new model outperforms the old model in terms of clinical diagnostic value, affirming the predictive worth of our prognostic model.

### 3.9 The analysis of the correlation between MANBA and M1 macrophages

**Figure 9:**
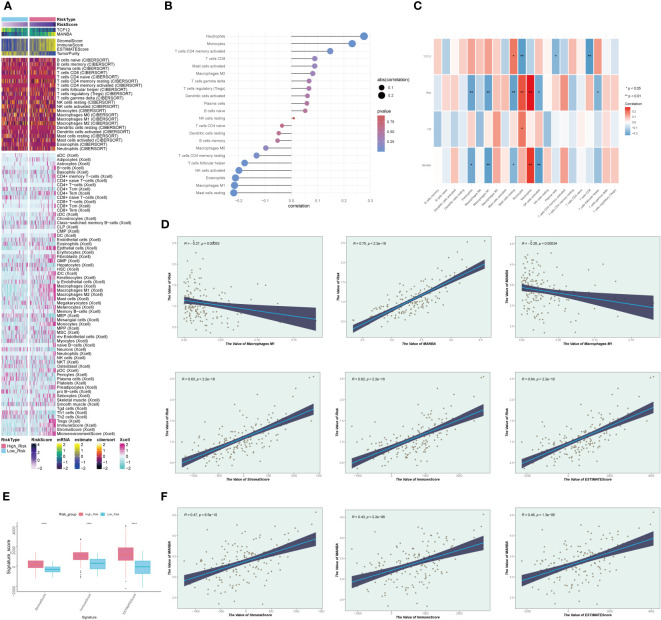
Immunocellular correlation analysis. **(A)**. Heatmap depicting differences in prognostic genes, tumor microenvironment, and immune cells between high and low-risk groups. **(B)**. Graph illustrating the expression relationship between immune cells and risk scores, with positive and negative decimals denoting positive and negative correlations, respectively. **(C)**. Diagram depicting the relationships between immune cells, prognostic genes, overall survival (OS), and risk scores, with red indicating positive correlations and blue indicating negative correlations. **(D)**. Scatterplot illustrating the correlations between MANBA, Macrophage M1, and risk scores. **(E)**. Boxplot of tumor microenvironment scores in high and low-risk groups. **(F)**. Scatterplot illustrating the correlations between tumor microenvironment scores, MANBA, and risk scores. *p<0.05; **p<0.01;****p<0.0001.

We observed significant differences in the distribution of the tumor microenvironment and M1 and M2 macrophages between the high and low-risk groups (**Figure 9A**). This piqued our interest, leading us to explore the correlation between different immune cell types and risk scores (**Figure 9B**). We found that M1 macrophages exhibited a negative correlation with risk scores, whereas M2 macrophages displayed a positive correlation, aligning with our earlier hypotheses. Furthermore, we delved into the relationships among risk scores, immune cells, and MR-DEGs (**Figure 9C**). Within M1 and M2 macrophages, only the correlation between M1 macrophages, risk scores, and MANBA was statistically significant. Based on this, we postulate that MANBA may inhibit the generation, transformation, and activity of M1 macrophages, thereby promoting tumor growth and adversely affecting the prognosis of GBM patients. Consequently, we analyzed the correlation between M1 macrophages, MANBA, and risk scores, illustrated in the scatter plot in **Figure 9D**. The results demonstrated a negative correlation between M1 macrophages and MANBA as well as risk scores, with statistical significance, in line with our hypotheses. We further investigated the differences in the tumor microenvironment between the high and low-risk groups (**Figure 9E**). We observed that the scores in the low-risk group were consistently lower than those in the high-risk group, with statistical significance. This leads us to speculate that the tumor microenvironment in GBM may provide a conducive milieu for tumor growth, with MANBA possibly playing a role therein. Building upon these findings, we conducted an investigation into the correlation between the tumor microenvironment and MANBA, as well as risk scores (**Figure 9F**). Our analysis revealed a positive correlation between stromal scores, immune scores, overall scores, and risk scores. Additionally, these scores displayed a positive correlation with MANBA, with statistical significance (P < 0.05), thus underscoring the meaningfulness of these results.

### 3.10 Functional enrichment analysis

**Figure 10:**
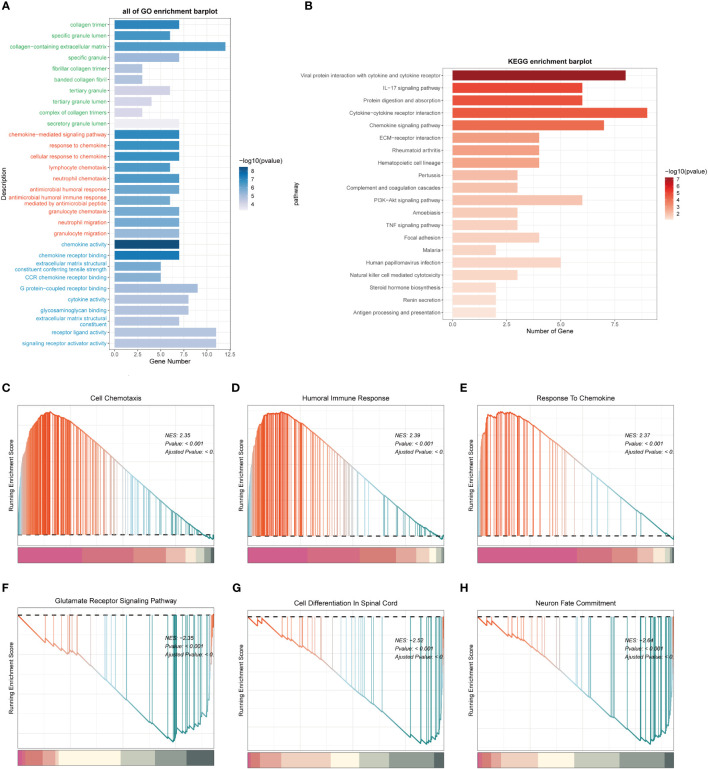
Functional enrichment analysis of differentially expressed genes in high and low expression groups. **(A)**. Functional Enrichment Analysis using GO terms. **(B)**. Functional Enrichment Analysis using KEGG pathways. **(C-E)**. Gene Set Enrichment Analysis (GSEA) for the High Expression Group. **(F-H)**. Gene Set Enrichment Analysis (GSEA) for the Low Expression Group.

In order to investigate the potential mechanisms and pathways associated with the expression of the MANBA gene, we selected genes that showed differential expression among patients in the high and low-risk groups. Subsequently, we subjected these genes to GO and KEGG enrichment analyses. GO analysis unveiled significant enrichment in pathways related to “collagen-containing extracellular matrix”, “chemokine-mediated signaling pathway” and “chemokine activity” among others (**Figure 10A**). Meanwhile, KEGG analysis demonstrated notable enrichment in pathways such as the “IL-17 signaling pathway” and the “Chemokine signaling pathway” (**Figure 10B**). We further subjected the gene sets of high and low-risk patient groups to Gene Set Enrichment Analysis (GSEA). Notably, we observed significant enrichments in the high-expression group for processes related to “Cell Chemotaxis,” “Humoral Immune Response,” and “Response To Chemokine,” (**Figures 10C-E**). Conversely, in the low-expression group, there were marked enrichments associated with “Glutamate Receptor Signaling Pathway,” “ Cell Differentiation In Spinal Cord,” and “Neuron Fate Commitment,” (**Figures 10F-H**).

### 3.11 Experimental validation of the role of the MANBA gene in GBM

**Figure 11:**
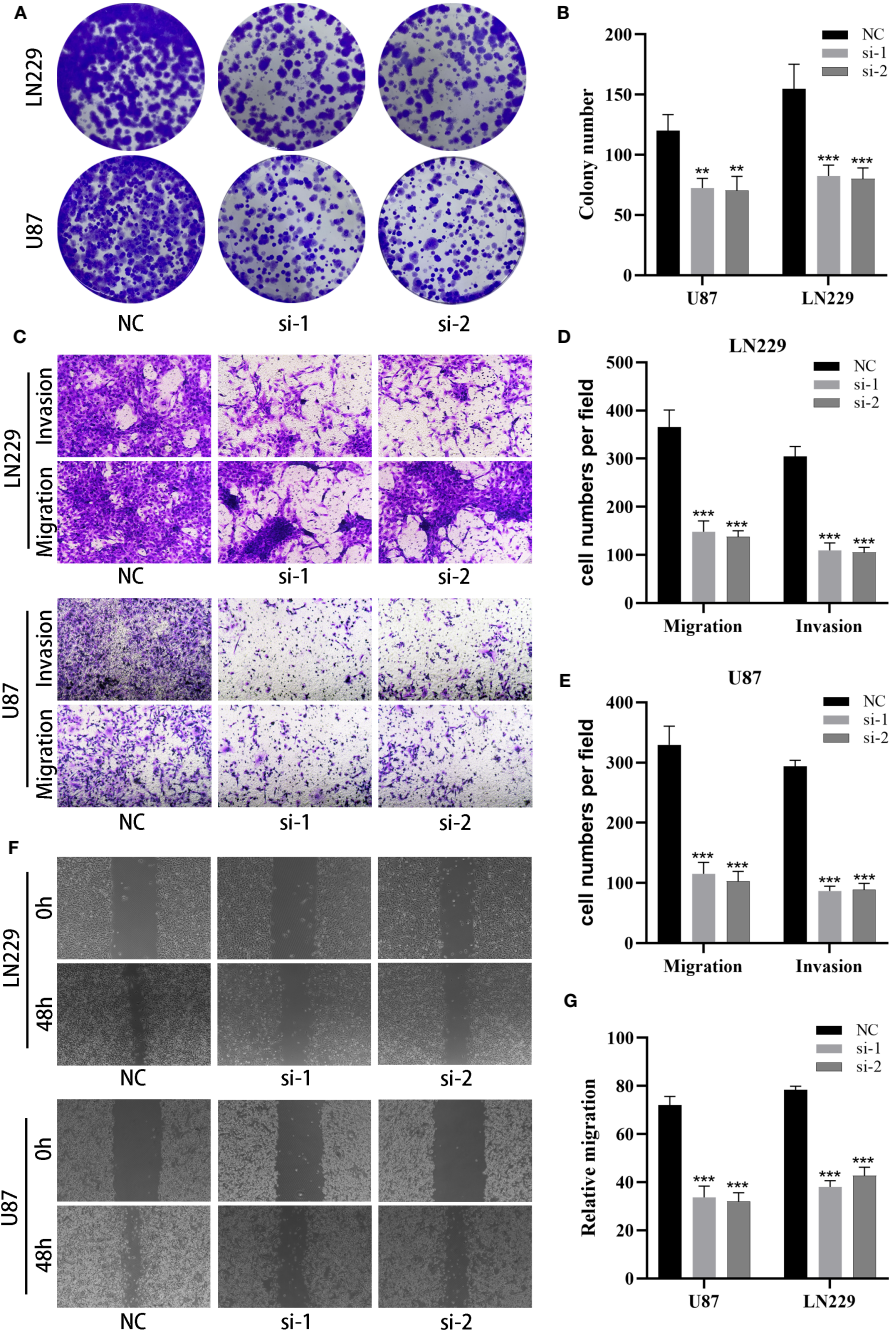
MANBA promotes malignant biological behavior of glioma cells. **(A, B)**. The effect of silencing MANBA on the proliferation of glioma was performed through colony formation assay **(A)** and relative quantification **(B)**. **(C-E)**. The effect of silencing MANBA on the migration and invasion ability of glioma was performed through transwell assay **(C)** and relative quantification **(D, E)**. **(F, G)**. The effect of silencing MANBA on the migration ability of glioma was performed through wound healing assay **(F)** and relative quantification **(G)**. **p<0.01; ***p<0.001.

Considering the involvement of MANBA in glioblastoma, we opted to study the effects of MANBA knockdown on two glioblastoma cell lines, namely U87 and LN229. Employing siRNA, we successfully reduced the expression of MANBA in these cell lines (**Figures 11A, B**). Subsequently, colony formation assays revealed a noteworthy decrease in the growth potential of the MANBA knockdown U87 and LN229 cells. Moreover, transwell assays confirmed the inhibitory effects of MANBA knockdown on tumor cell migration. Furthermore, scratch assays demonstrated a significant reduction in the invasive capability of glioblastoma cells upon MANBA inhibition (**Figures 11C-E**). Accordingly, we proceeded with further experiments to validate the role of MANBA in fostering the proliferation, invasion, and metastasis of glioblastoma cells (**Figures 11F, G**).

## 4 Discussion

Glioblastoma multiforme (GBM) is a prevalent primary malignancy affecting the nervous system, with an estimated annual mortality rate of up to 10,000 individuals in the United States (www.braintumor.org). Approximately 81% of malignant brain tumors are caused by GBM, despite surgical resection, radiotherapy, and chemotherapy, patients with GBM have a mean survival time of only 14.6 months (24, 25). In theory, immunotherapy may represent a promising treatment option for GBM due to the ability of immune cells to cross the blood-brain barrier and target GBM cells (26). However, GBM is considered an immune cold tumor due to the cellular composition of macrophages and microglia in the surrounding microenvironment, resulting in limited efficacy of many immunotherapies (23, 27). In light of these challenges, we conducted a secondary analysis of data from Xie et al. (60), integrating cell trajectory and communication analyses to identify potential mechanisms underlying the effects of macrophages and microglia on GBM. Moreover, we aimed to establish a prognostic model based on these findings.

Through the annotation of cells in the tumor core tissue and peritumoral tissue, we have found that the macrophage distribution is concentrated in the tumor core tissue. This has aroused our interest. We have therefore further contrasted the activity of oxidative stress pathways in macrophages and other subsets. Oxidative stress response is actually a double-edged sword in terms of tumors, which may either lead to cellular genetic mutations or inhibit the immune response, thus promoting tumor growth. Excessive ROS in turn may induce the tumor cell death and enhance their sensitivity to chemotherapy (28–30). Through three computational methods, we have found that the oxidative stress score of macrophages is higher than that of the other subsets. Moreover, the macrophages are tumor core cells, which seems to indicate that oxidative stress plays a side effect in GBM. Kuo (31) et al. have concluded that the activation of ROS is often accompanied by vascular proliferation, inhibition of immune microenvironment function and polarization of M1 to M2 cells. These functions will greatly promote the progress of GBM. In addition, the ROS may also induce the secretion of extracellular carriers and further enhance the production of IFN and IL-6 in macrophages, thereby inhibiting the immune response in the tumor microenvironment (21, 32, 33). Griess (34) et al. have discovered that ROS is the best second messenger required for M2 polarization in the IL-4 signaling pathway. Eliminating ROS by oxidation-reduction drugs may selectively inhibit the polarization of M2 and promote the tumor growth. However, reactive oxygen species (ROS) can also induce lipid peroxidation or disrupt intracellular proteins and nucleic acids, leading to cell necrosis or apoptosis (61). Research has identified several associated pathways; for example, Guan et al. discovered that ROS can activate the Fas/FasL pathway, leading to caspase activation and inducing cell apoptosis (62). Similarly, Angkeow et al. found that activated NF-κB can bind to the corresponding DNA, promoting the transcription of target genes and thereby inducing cell apoptosis (63). Likewise, in GBM, elevated ROS levels can also lead to mitochondrial apoptosis, thereby causing the demise of GBM cells (35). Therefore, we speculate that the oxidative stress pathway is closely related to macrophage polarization and inhibition of immune function in GBM. By a moderate modulation of the degree of oxidative stress response, it may induce the mitochondrial apoptosis to kill tumor cells as well as prevent the excessive polarization and immunosuppressive activities of M2. This is a good research direction.

By comparing the proportion of each cell subset in GBM, we have found that macrophages account for the largest proportion in the tumor core tissue. It is suspected that this may be correlated with some signaling molecules. Therefore, we have further explored the cell interaction signals between the macrophages and GBM subsets, and found that SPP1-CD44 is not only interacted significantly among the macrophages, but also interacted significantly between the macrophages and other tumor core cells such as T cells and dendritic cells. Secreted phosphoprotein 1 (SPP1) is also called osteopontin (OPN). It is a multifunctional cytokine, which is found to be highly expressed in a variety of cancers (36–38). It is positively associated with the poor prognosis of GBM (39). SPP1 factors also interact with CD44 receptor to activate downstream signaling pathways and regulate cell adhesion, tumor progression and metastasis of (40, 41). Interestingly, M2-like macrophages associated with SPP1 are discovered in a variety of cancers and located in the tumor core tissue (42, 43). Moreover, Zhang (44) et al. have found that SPP1 upregulation promotes the polarization of M1 to M2 in macrophages. Whereas, M1 to M2 transformation mostly predicts GBM progression and poor prognosis. Furthermore, CD8+ cytotoxic T cells are the primary immune cells used to eradicate tumors (45, 46). While the process activated from naive T cells to CD8+ cytotoxic T cells may be inhibited by SPP1 ligands (47), thus causing immunosuppression. However, our study reveals that the SPP1-CD44 interaction of macrophages is especially significant in GBM. We therefore hypothesize that SPP1-CD44 is associated with immunosuppression of the GBM microenvironment. The SPP1-CD44 interaction among the macrophages could promote the transformation of M1 to M2, resulting in a poor prognosis in patients. This is consistent with the results of He (48) et al. However, we have also found a strong SPP1-CD44 interaction between the macrophages and T cells in GBM. We believe it is also one of the key points for GBM progression and it can exist as a key target for therapy.

Alternatively, GBM can attract macrophages to aggregate and activate (49) by producing cytokines, and together with other subsets to constitute an immune microenvironment of GBM. In this microenvironment, macrophages are forced to intensify from M1 to M2 and secrete cytokines such as IL-10, macrophage colony stimulating factors, TGF- β to help tumors escape (15, 50). This may be why there are trajectories within macrophages. Its excretory factors such as IL-10 may also inhibit the proliferation of T cells and enhance the activity of regulatory T cells (Treg), thus playing a role of immunosuppression (51, 52). At the same time, we have studied the evolution process of each cell subset in GBM and found that the macrophages are located in the center of the trajectory through the analysis results of the three methods. They are related to all cell subsets in GBM. This indicates that macrophages are the core of each cell subset in GBM, and their functional activities may affect the surrounding subsets and strengthen immunosuppression, thereby affecting the trend of the entire GBM. If the aggregation of macrophages can be avoided and their polarization process is reversed, it should have a positive effect on preventing the generation of the inhibitory immune microenvironment and improving the immune function of the surrounding subsets.

We have further explored the prognostic genes associated with macrophages. Through analysis, we have finally identified two genes, including the high-risk gene MANBA and the low-risk gene TCF 12. MANNBA is a gene encoding ß -mannosidase. A gene deficiency may cause β -mannoside storage disease, which can be accompanied by a wide range of neurological symptoms (53). In some studies, the role of MANBA in cancer has been reported, such as being involved in the occurrence and metastasis of colorectal cancer and human esophageal squamous cell carcinoma (54, 55). Wielgat (56) has discovered that the glycosidase activity is positively correlated with the malignancy of glioma, which is consistent with our findings. In addition, some studies have found that MAMBA is associated with neutrophil degranulation and may affect the activity of lymphocytes (57–59).In addition, We observed a significant negative correlation between M1 macrophages, risk scores, and MANBA expression, highlighting substantial distinctions. This not only validates the favorable impact of M1 macrophages on the prognosis of GBM patients but also elucidates that heightened MANBA expression results in the inhibition of M1 macrophage generation, transformation, and activity. Consequently, we hypothesize that MANBA potentially engages in functional regulation, suppressing the generation and activation of M1 macrophages while comparatively promoting M2 transformation. This suggests an immunosuppressive effect, thereby fostering tumor proliferation and progression.

Furthermore, we carried out experimental validations to confirm the role of MANBA in GBM, and the results were consistent with our initial hypotheses. Knocking down MANBA had a suppressive effect on the proliferation, migration, and invasion capabilities of GBM cells. This underscores the significant role of MANBA in the progression of GBM, potentially involving immune functions such as the inhibition of M1 macrophage transformation and activity. However, the specific mechanisms remain incompletely understood, necessitating further experimental verification. In conclusion, our findings anticipate a pivotal role for macrophages in GBM, uncovering key pathways and signaling molecules that influence macrophage transformation. Additionally, we provide critical prognostic genes associated with these processes, offering new research and therapeutic directions for targeted immunotherapy and prognosis diagnosis of GBM in the future. However, this study has some limitations. The overall sample size was limited, and results may be influenced by certain incidental factors. Furthermore, the single-cell samples analyzed primarily originated from CD31+ endothelial cells in GBM, making it challenging to encompass all cell types within GBM. In subsequent investigations, we aim to enhance our research outcomes by analyzing a more extensive array of single-cell samples. We also plan to refine associated experiments to delve into the specific immunological mechanisms involving the MANBA gene. Addressing these limitations is a priority in our future research endeavors.

## 5 Conclusion

In summary, our study has comprehensively characterized the different cell subpopulations present in both the GBM core and surrounding tissues. We have investigated the relationship between macrophages and GBM and their associated mechanisms. Our findings reveal that macrophages play a critical role in the trajectory of each cell subset within GBM. We have examined the oxidative stress response in macrophages and investigated the immunosuppressive effects of interaction signals such as SPP1-CD44 on macrophage polarization and the cell subsets in the GBM tumor microenvironment. Additionally, we have identified the macrophage-related prognostic genes MANBA and TCF12. Furthermore, we postulate that MANBA assumes a critical role in promoting GBM progression, potentially by being involved in immunosuppressive functions through the inhibition of M1 macrophage generation and transformation. Our experimental validation of MANBA’s enhancement of GBM proliferation, invasion, and metastatic capabilities indirectly corroborates our conjectures. In conclusion, our research delves into novel immunological mechanisms within GBM, thereby providing insights into the identification of new prognostic markers and immunotherapeutic targets for GBM.

## Data availability statement

The data for this study come from the Gene Expression Omnibus (GEO) (https://www.ncbi.nlm.nih.gov/geo/) database and the Cancer Genome Atla s database (https://portal.gdc.cancer.gov/). The GEO accession is GSE162631. All the data in this paper support the results of this study.

## Author contributions

JX: Conceptualization, Data curation, Formal analysis, Investigation, Methodology, Project administration, Resources, Software, Supervision, Validation, Visualization, Writing – original draft, Writing – review & editing. HC: Investigation, Methodology, Resources, Visualization, Writing – original draft. ZL: Data curation, Software, Visualization, Writing – original draft, Writing – review & editing. HC performed the experimental part of the paper. LZ: Formal analysis, Investigation, Methodology, Writing – original draft. HX: Conceptualization, Data curation, Methodology, Writing – review & editing. YS: Investigation, Methodology, Project administration, Writing – original draft. ZHW: Conceptualization, Investigation, Software, Writing – original draft. CL: Methodology, Project administration, Validation, Writing – review & editing. GH: Data curation, Software, Validation, Writing – original draft. JZ: Methodology, Writing – original draft. LR: Data curation, Investigation, Writing – original draft. ZLW: Funding acquisition, Project administration, Resources, Supervision, Validation, Writing – review & editing.

## Supplementary material

Supplementary Figure 1 Results of each sample after removal of double cells using R package DoubletFinder.

Supplementary Figure 2 Basic Single Cell Transcriptome Analysis Process. **(A)**. The result of each sample quality control after filtering. **(B)**. Cell cycle display. **(C)**. Highly variable gene display. **(D)**. Standard Deviation under different PCs. **(E)**. The distribution of different samples under 2 PCS.
