# Supplementary figures and images for: Examining the function of macrophage oxidative stress response and immune system in glioblastoma multiforme through analysis of single-cell transcriptomics
Source: Front Immunol. 2024 Jan 11;14:1288137. doi: 10.3389/fimmu.2023.1288137 (PMC10808540; doi:10.3389/fimmu.2023.1288137)

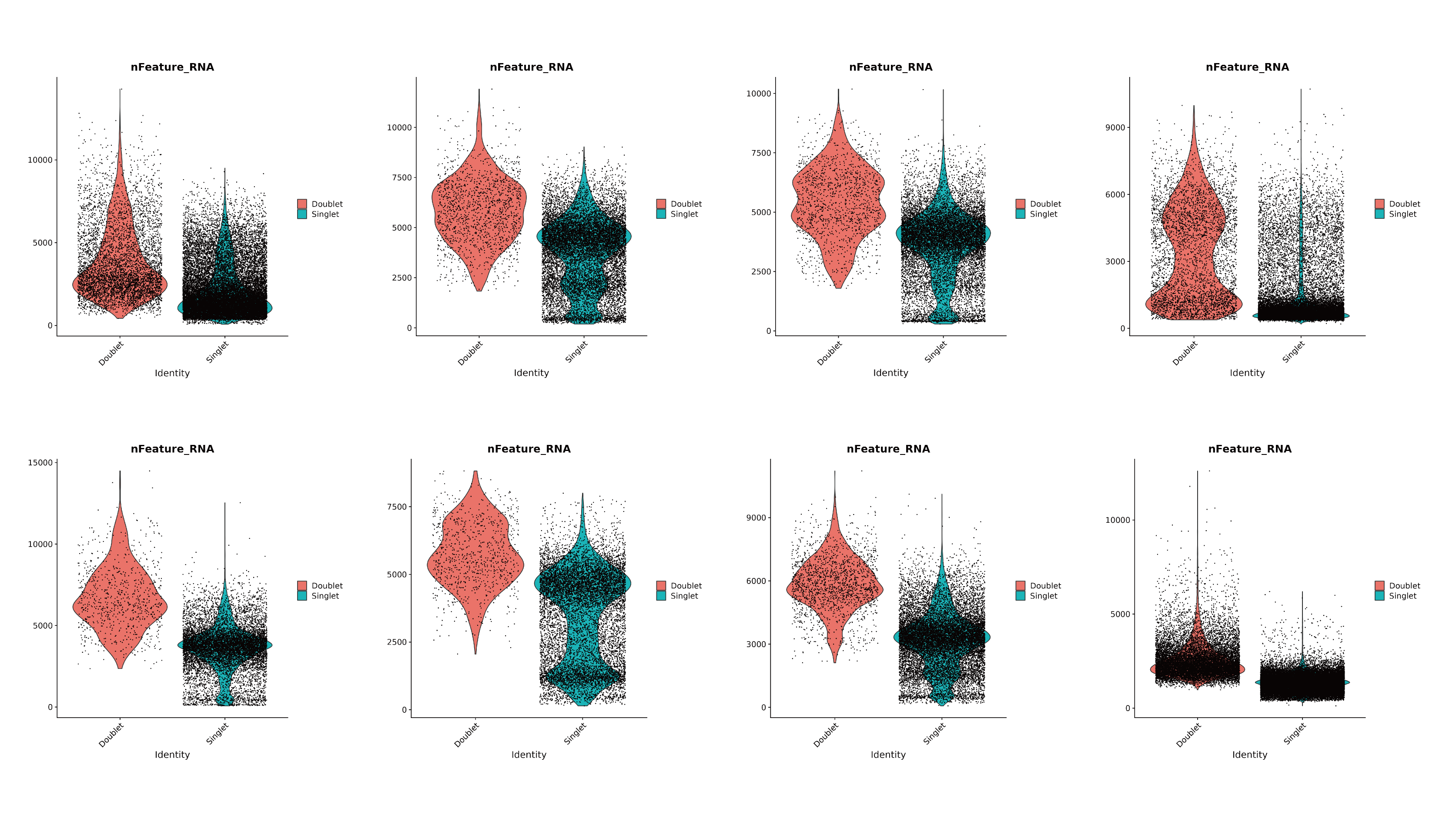

Supplement: Supplementary Figure 1 — Results of each sample after removal of double cells using R package DoubletFinder. [file Image_1.tif]

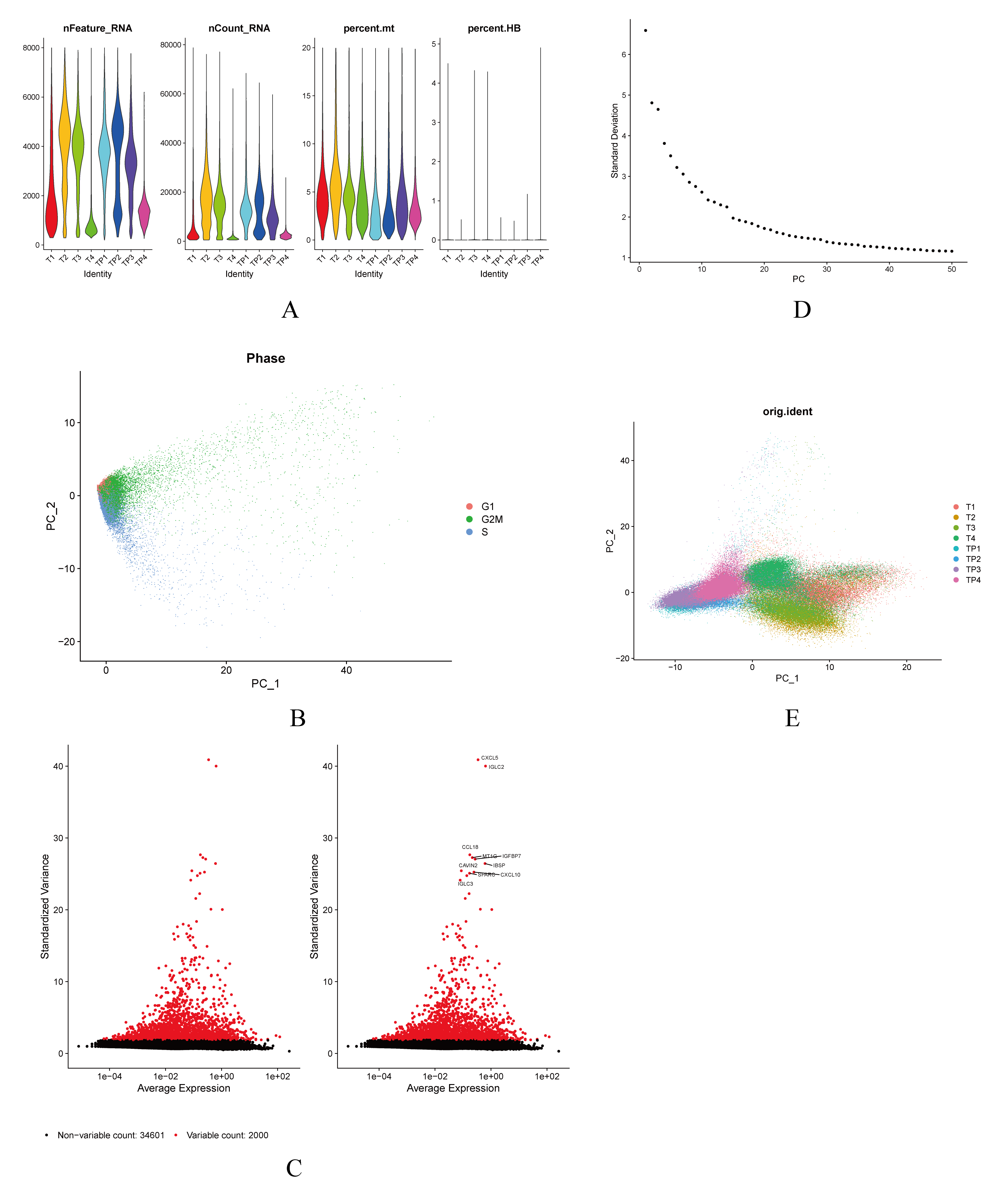

Supplement: Supplementary Figure 2 — Basic Single Cell Transcriptome Analysis Process. (A). The result of each sample quality control after filtering. (B). Cell cycle display. (C). Highly variable gene display. (D). Standard Deviation under different PCs. (E). The distribution of different samples under 2 PCS. [file Image_2.tif]
